# Supplementary material for: Widespread and complex drought effects on vegetation physiology inferred from space
Source: Nat Commun. 2023 Aug 15;14:4640. doi: 10.1038/s41467-023-40226-9 (PMC10427636; doi:10.1038/s41467-023-40226-9)
Supplement: Supplementary file 1 — Supplementary Information File [file 41467_2023_40226_MOESM1_ESM.pdf]

Supplementary figures

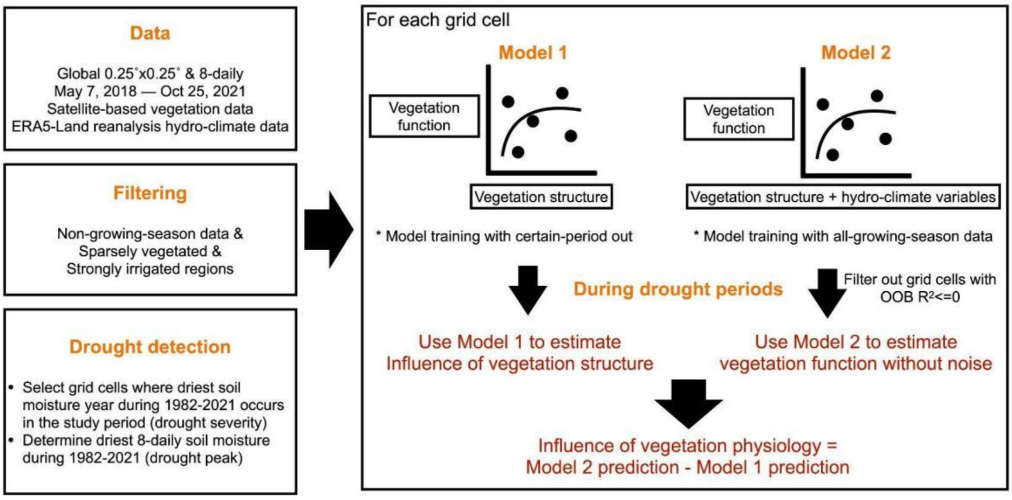

Figure S1. The flow chart of data processing and methodology for disentangling physiological components from the vegetation observations and observation-derived products. Details about data-processing can be found in the Method section (Data pre-processing; Data pre-processing). When disentangling vegetation physiological components, two types of models are implemented: Model 1 with LAI as the only predictor is trained using the leave-out strategy which can help to overcome an over-fitting-related overestimation of structural influence on predictions. The leave-out strategy is applied for removing 6, 12, 24 time-step data (each time step is 8 days) to test the method robustness. Model 1 can then be used to infer the influence of vegetation structure; Model 2 includes LAI and hydro-climate variables (temperature, solar radiation, precipitation, soil moisture, and vapor pressure deficit) as predictors to predict vegetation observations (i.e. SIFrel, ET, and VOD ratio) while removing potential data noise. The difference between the predicted vegetation overall signals and vegetation structural signals are hence the vegetation physiological signals. We apply the disentangling method for each grid cell and for each vegetation functional related indices.

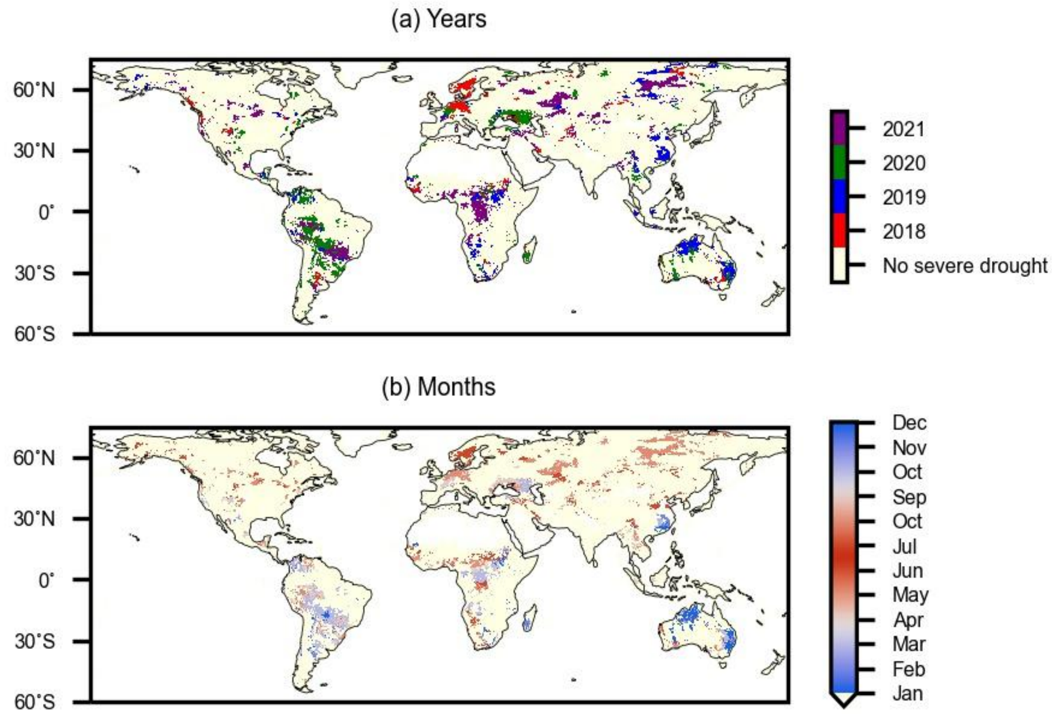

Figure S2. (a) Years and (b) months of drought peaks as detected by soil moisture minima during growing seasons defined for each grid cells within the study period March 2018–October 2021. Grid cells without severe drought events in this period are disregarded.

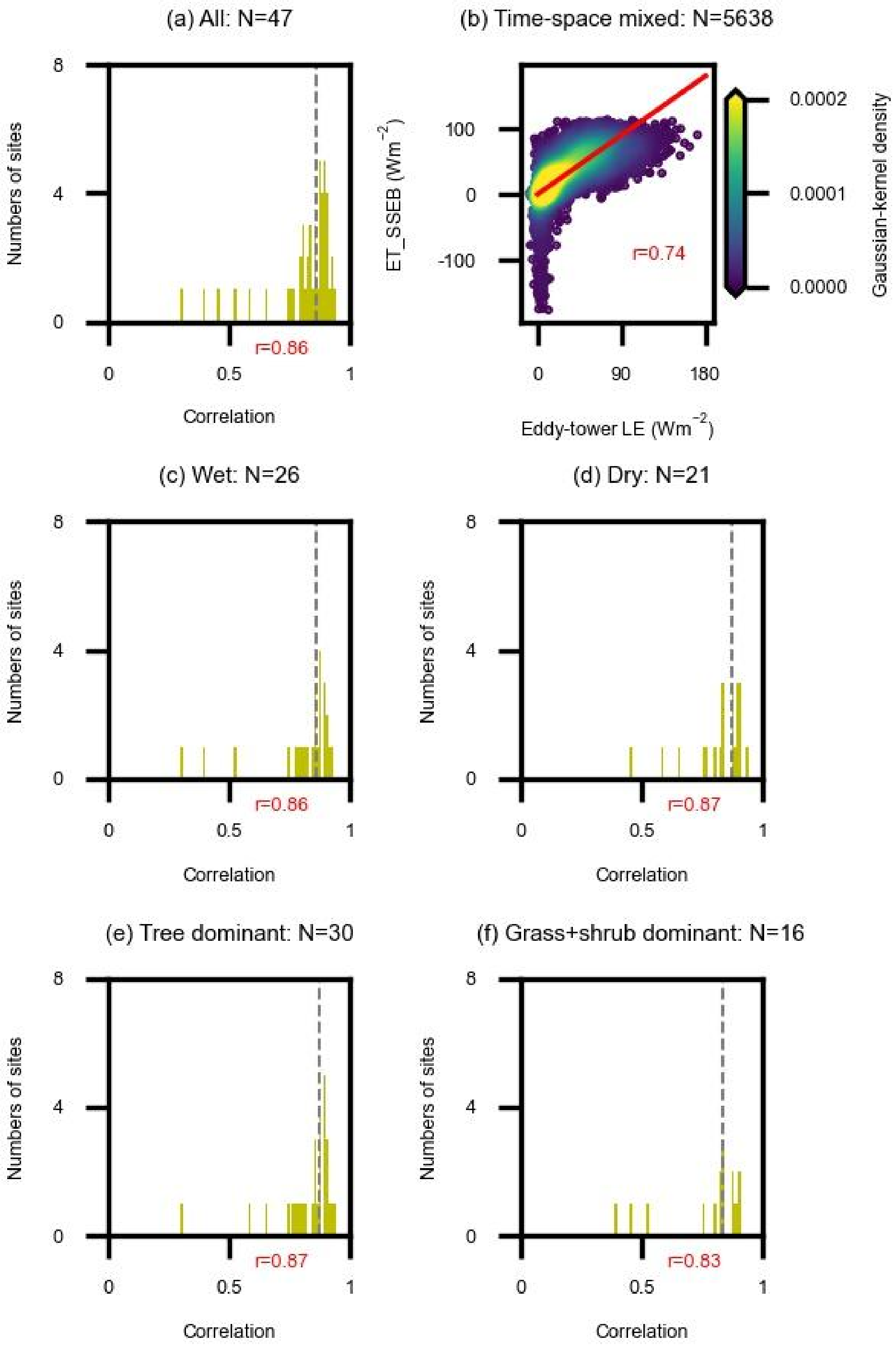

Figure S3. Comparisons between SSEB-based and eddy covariance-measured ET for the whole growing season. (a, c-f) The distribution of correlation between SSEB-based ET and eddy-tower LE for all sites and sub-sites distinguished by aridity and tree/(grass+shrub) ratios. Grey dashed lines and red texts denote the median values of correlation coefficients across sites. (b) The distribution of SSEB-based ET and eddy-tower LE values corresponding to each site and time step.

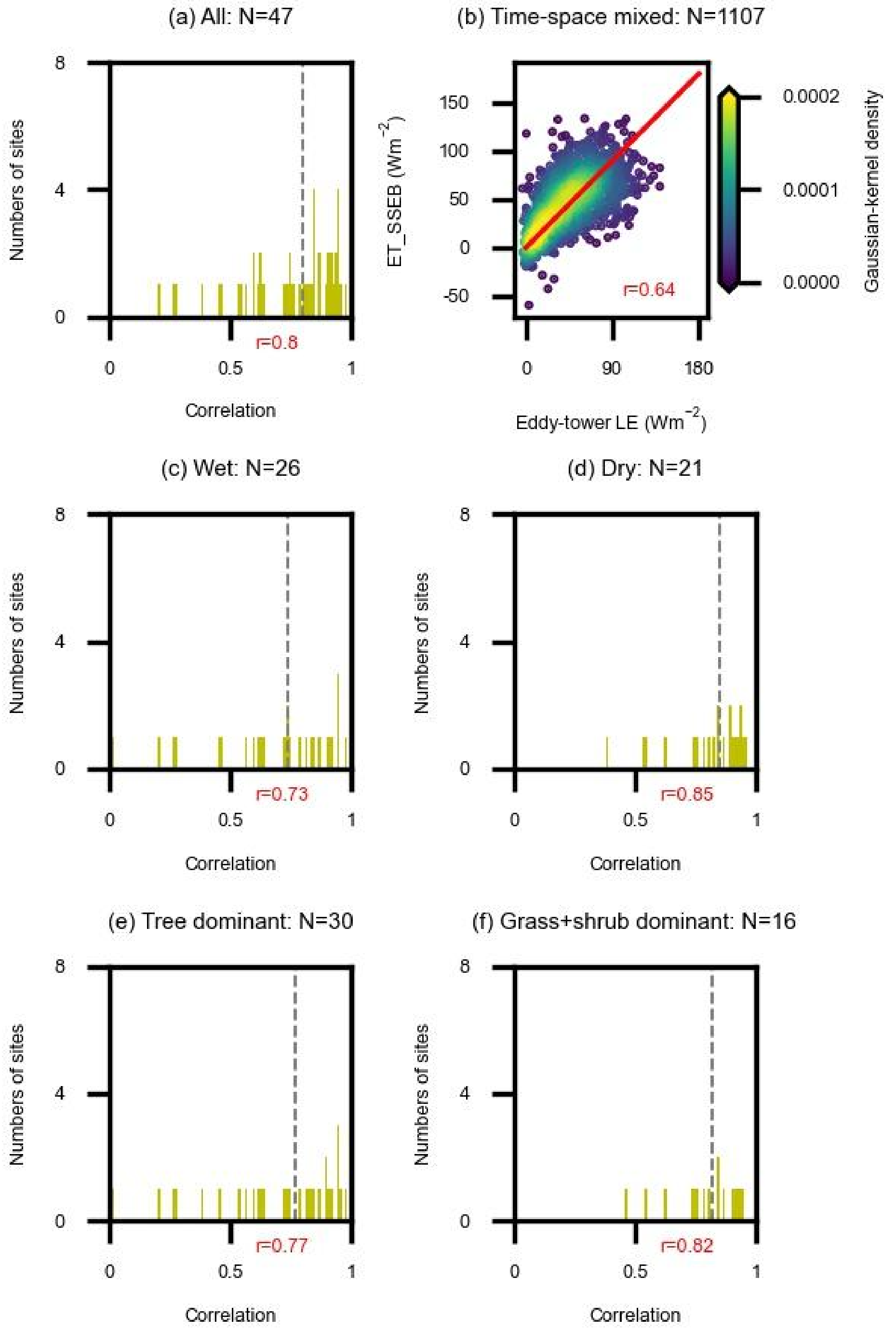

Figure S4. Similar as in Fig. S3 but for 6-month drought periods defined by gridded soil moisture data. (a, c-f) The distribution of correlation between SSEB-based ET and eddy-tower LE for all sites and sub-sites distinguished by aridity and tree/(grass+shrub) ratios. Grey dashed lines and red texts denote the median values of correlation coefficients across sites. (b) The distribution of SSEB-based ET and eddy-tower LE values corresponding to each site and time step.

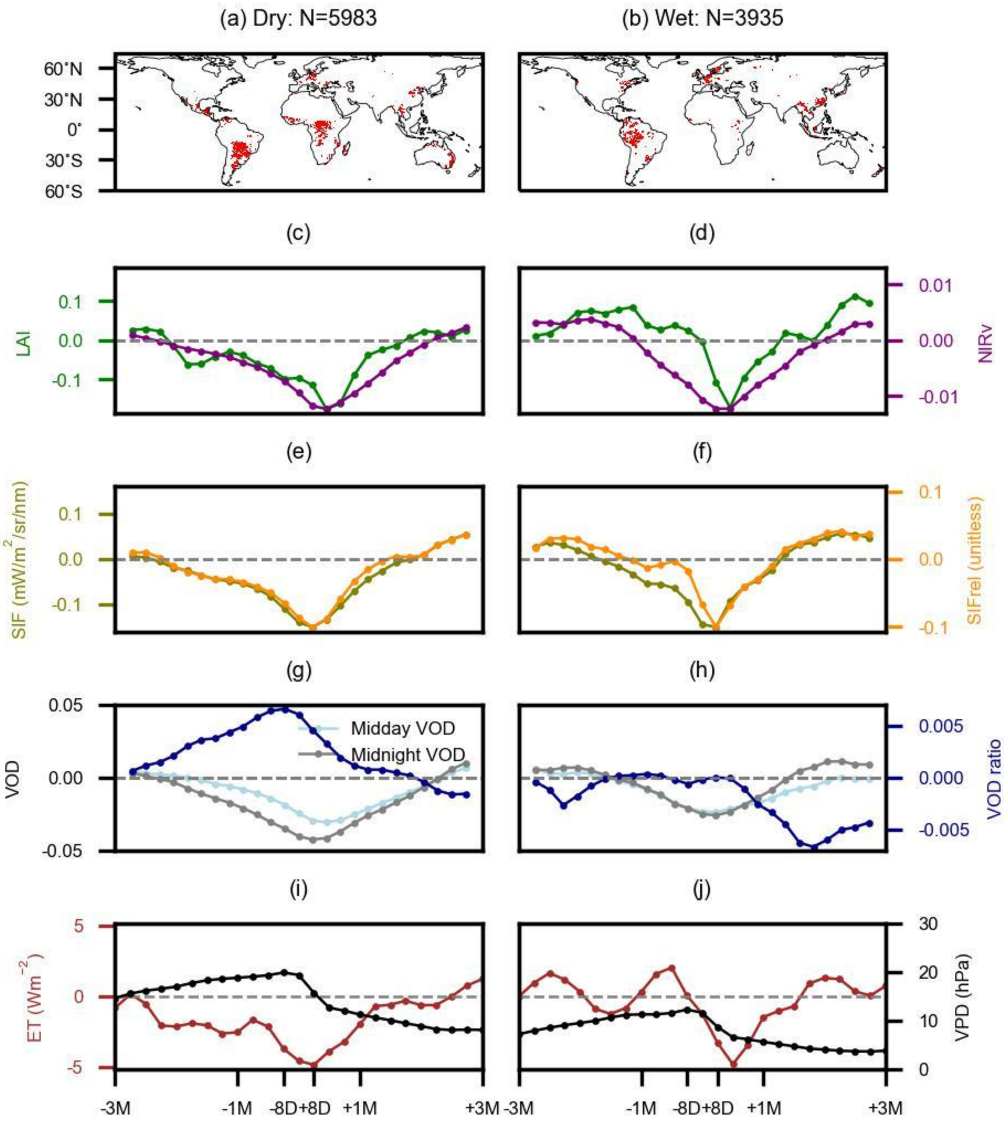

Figure S5. Same as in Fig. 2 but replacing absolute soil moisture by VPD. Drought-affected grid cells in (a) dry regions (aridity > 1) and (b) wet regions (aridity <= 1). Results for dry regions are presented in (c, e, g, i) and for wet regions are presented in (d, f, h, j). (c, d) LAI and NIRv, (e, f) SIF and relative SIF (SIFrel), (g, h) VOD at midday, midnight, and the ratio between them (VOD ratio), (i, j) ET and VPD. All vegetation variables are shown as anomalies, except for VPD in (i, j) which is presented in absolute values to indicate the actual VPD amount.

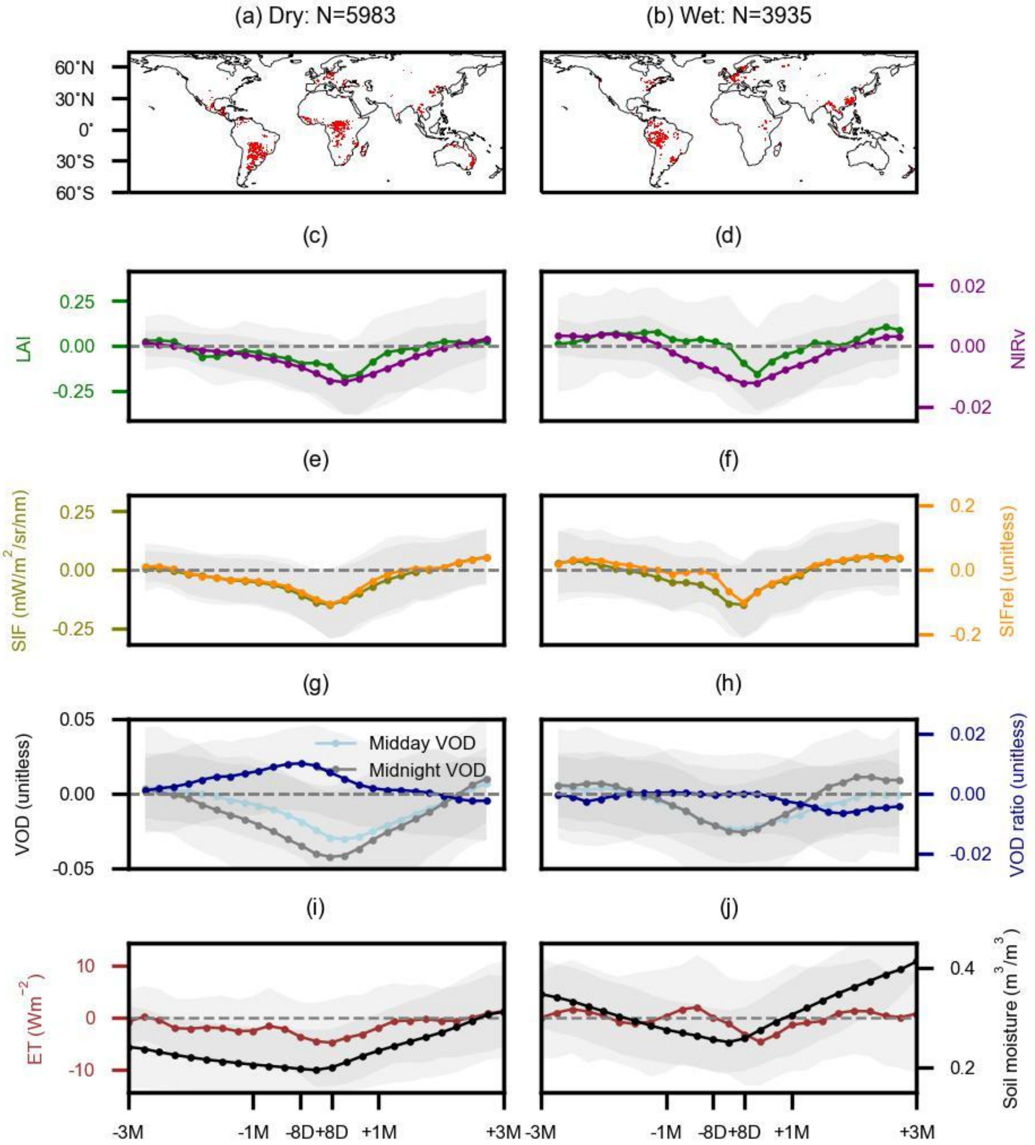

Figure S6. Same as in Fig. 2 but with inter-quartile ranges in the grey shade. Drought-affected grid cells in (a) dry regions (aridity > 1) and (b) wet regions (aridity <= 1). Results for dry regions are presented in (c, e, g, i) and for wet regions are presented in (d, f, h, j). (c, d) LAI and NIRv, (e, f) SIF and relative SIF (SIFrel), (g, h) VOD at midday, midnight, and the ratio between them (VOD ratio), (i, j) ET and soil moisture. All vegetation variables are shown as anomalies, except for soil moisture in (i, j) which is presented in absolute values to indicate the actual water amount. The solid lines denote mean values across grid cells. Shades in figures denote inter-quartile (25 - 75 %) ranges.

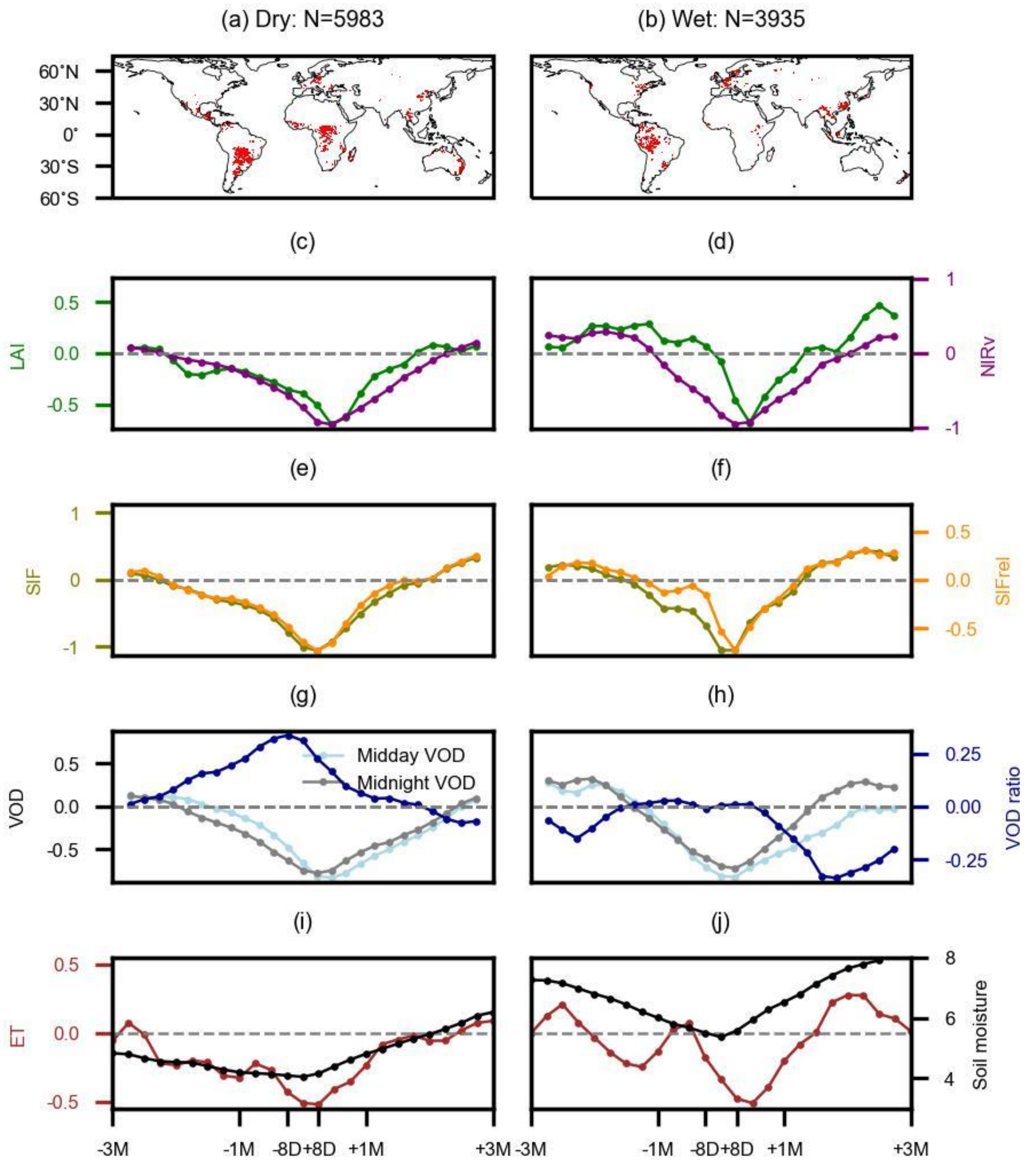

Figure S7. Similar to Fig. 2 but presenting the normalised vegetation anomalies using the anomalies divided by respective standard deviation for each grid cell. Drought-affected grid cells in (a) dry regions (aridity > 1) and (b) wet regions (aridity <= 1). Results for dry regions are presented in (c, e, g, i) and for wet regions are presented in (d, f, h, j). (c, d) LAI and NIRv, (e, f) SIF and relative SIF (SIFrel), (g, h) VOD at midday, midnight, and the ratio between them (VOD ratio), (i, j) ET and soil moisture. Soil moisture absolute values are also divided by their standard deviation.

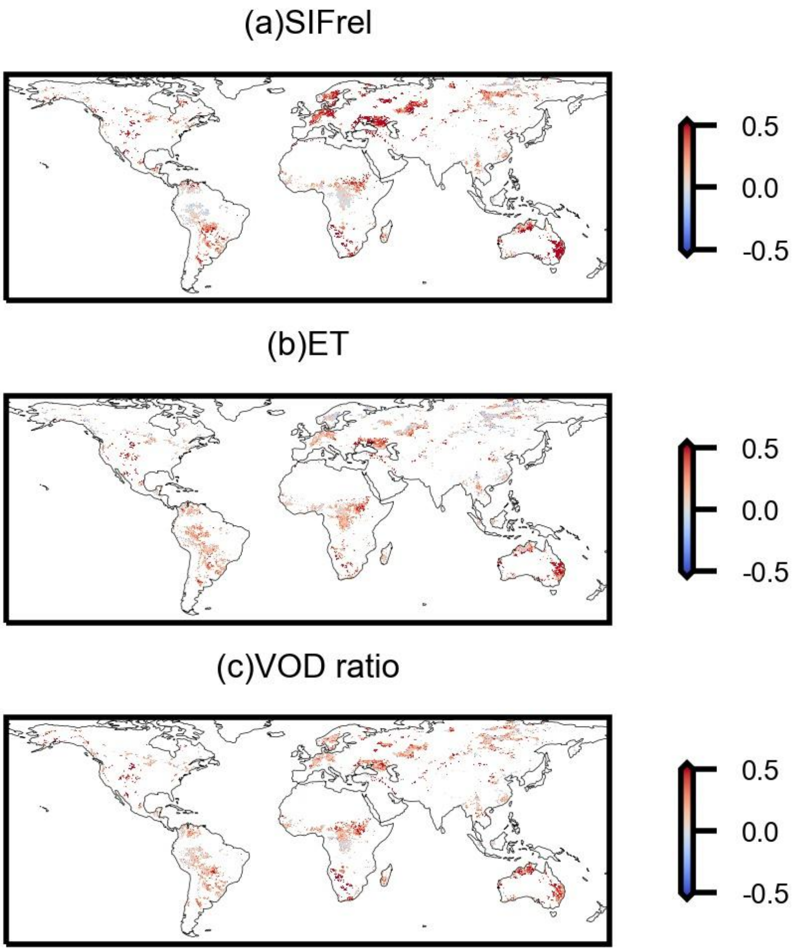

Figure S8. The out-of-bag R^2 score from the cross-validation of random forests including LAI and hydro-meteorological data as inputs to predict (a) SIFrel, (b) ET, and (c) VOD ratio.

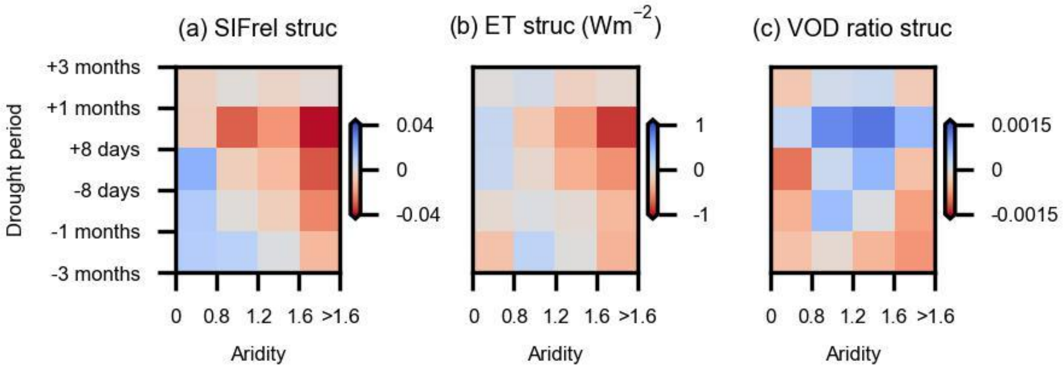

Figure S9. Similar to Fig.3 (d-f) but for changes related to vegetation structural changes as estimated from LAI for (a) SIFrel, (b) ET, and (c) VOD ratio.

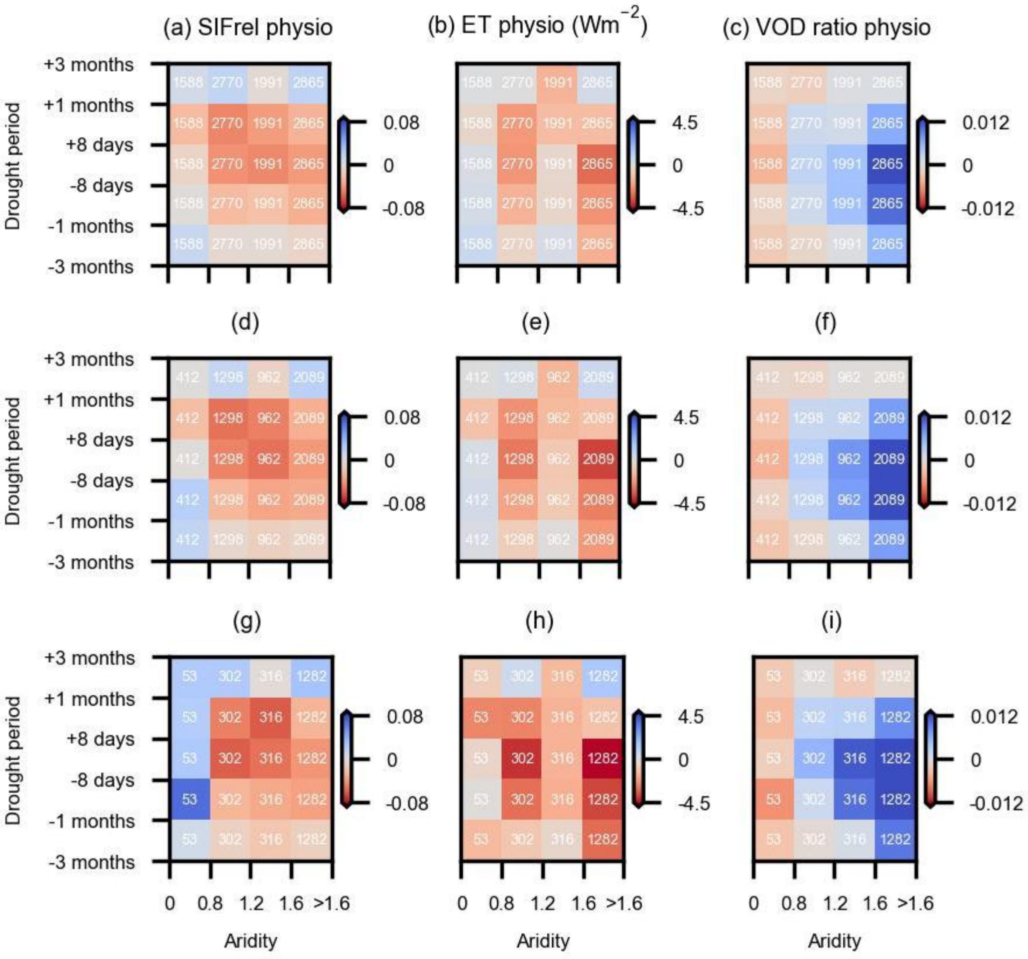

Figure S10. (a-c) Same as Fig.3 (d-f) with white numbers denoting numbers of grid cells belonging to each aridity group. (d-i) Similar to Fig.3 (d-f) but keeping regions with random forest out-of-bag $R^2 > 0.1$ (a-f) and out-of-bag $R^2 > 0.2$ (g-i).

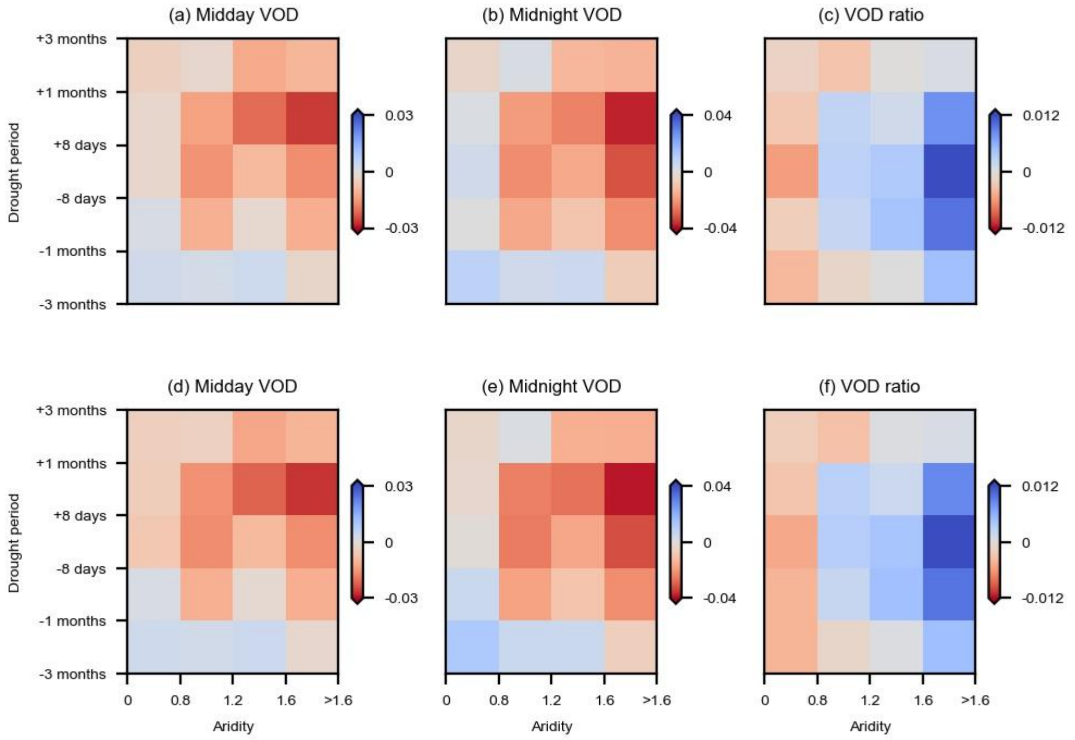

Figure S11. Anomalies of midday, midnight VOD, and VOD ratio during droughts across aridity (a, b, c) before and (d, e, f) after removing regions with incomplete growing-season root-zone water refilling at 1:30am.

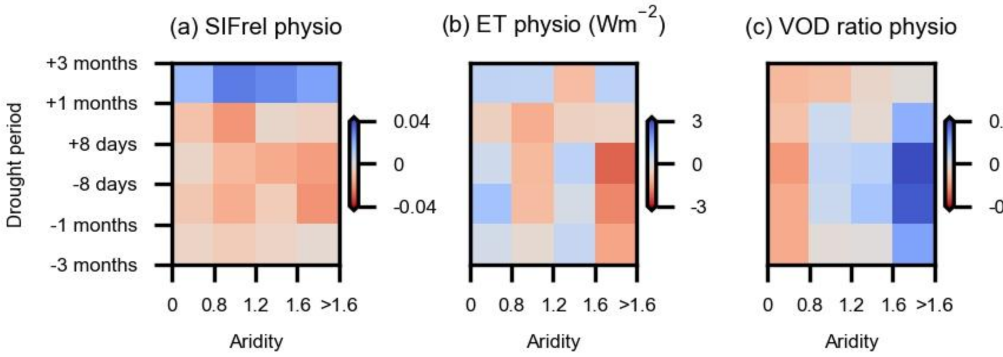

Figure S12. Vegetation physiological controls on (a) SIFrel, (b) ET, and (c) VOD ratio by using NIRv to reflect vegetation structure, since it can test result robustness of using LAI and it additionally accounts for the fluorescence escape probability.

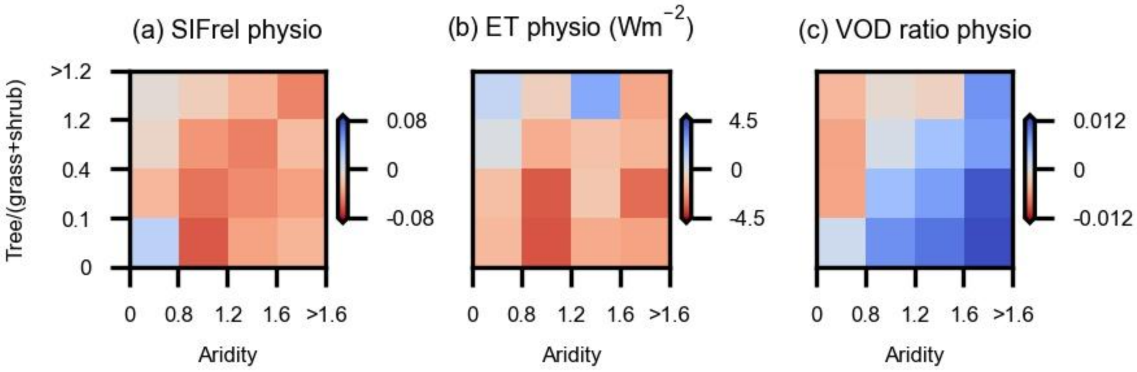

Figure S13. (a) SIFrel, (b) ET, and (c) VOD ratio physiological responses to drought across aridity and vegetation types during 8-day before drought until drought peaks. Vegetation types are represented by the ratio between the tree cover fraction and the cover fraction of grass and shrub.

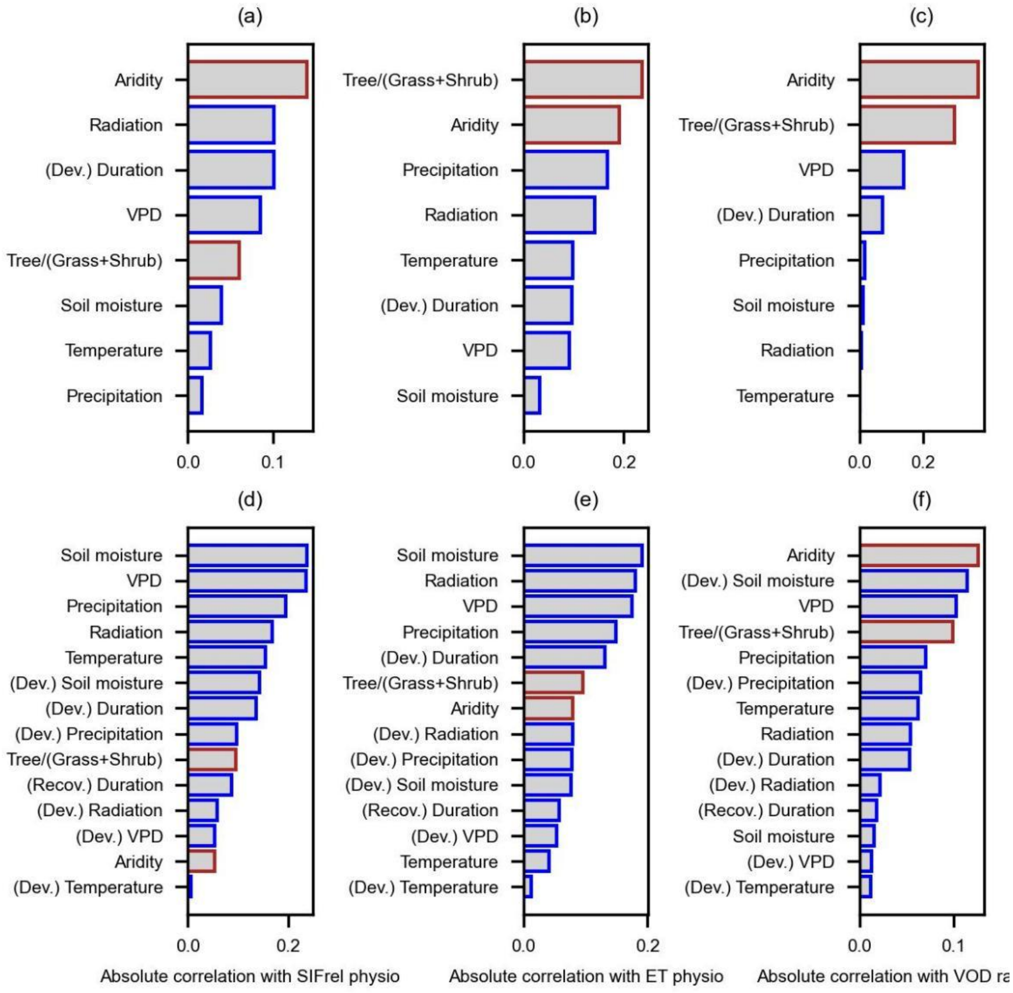

Figure S14. Similar to Fig. 4 but using spearman correlation for each predictor and vegetation physiological variable to determine variable importance ranking. Results show their relevance (with the same unit of target variables) in explaining the spatial variability of anomalies in (a) SIFrel physiology (SIFrel physio, unitless), (b) ET physiology (ET physio, Wm^{-2}) and (c) VOD ratio (unitless) during drought development. (d-f) Similar as in (a-c) but for drought recovery periods where we consider drought-development (Dev.) and recovery (Recov.) related drought duration and hydro-meteorological anomalies. The unit of relative importance is the same for each physiological variable. Radiation refers to incoming shortwave radiation.

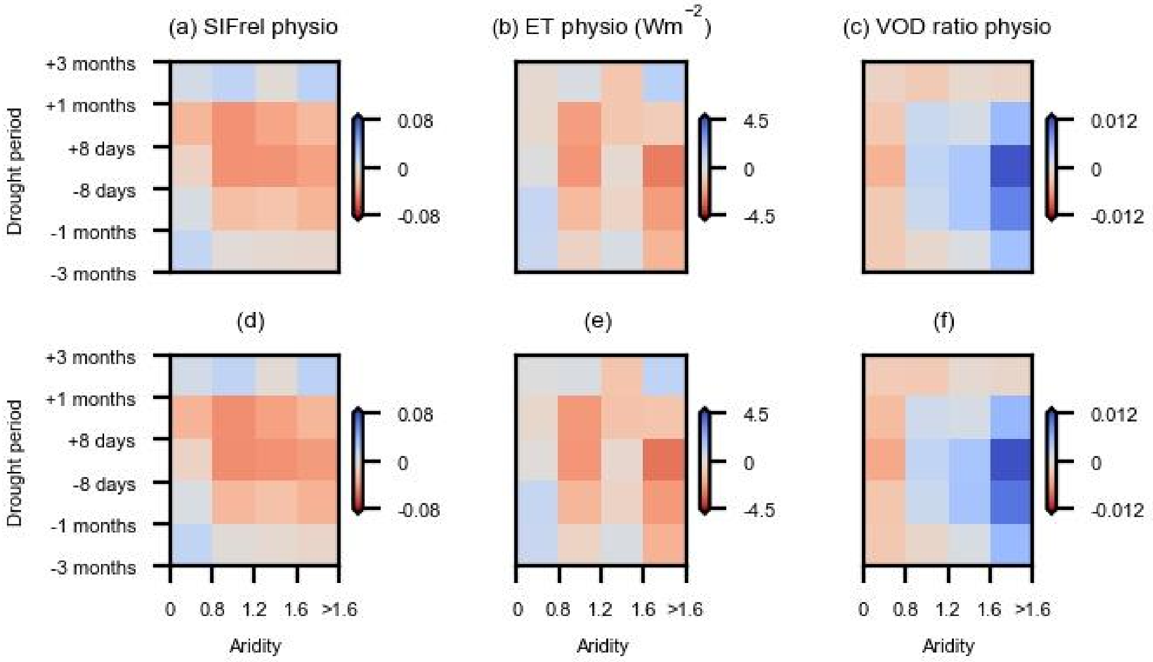

Figure S15. Same as in Fig.3 (d-f) but leaving out (a, b, c) 12 time steps or (d, e, f) 6 time steps to train random forest models by LAI.

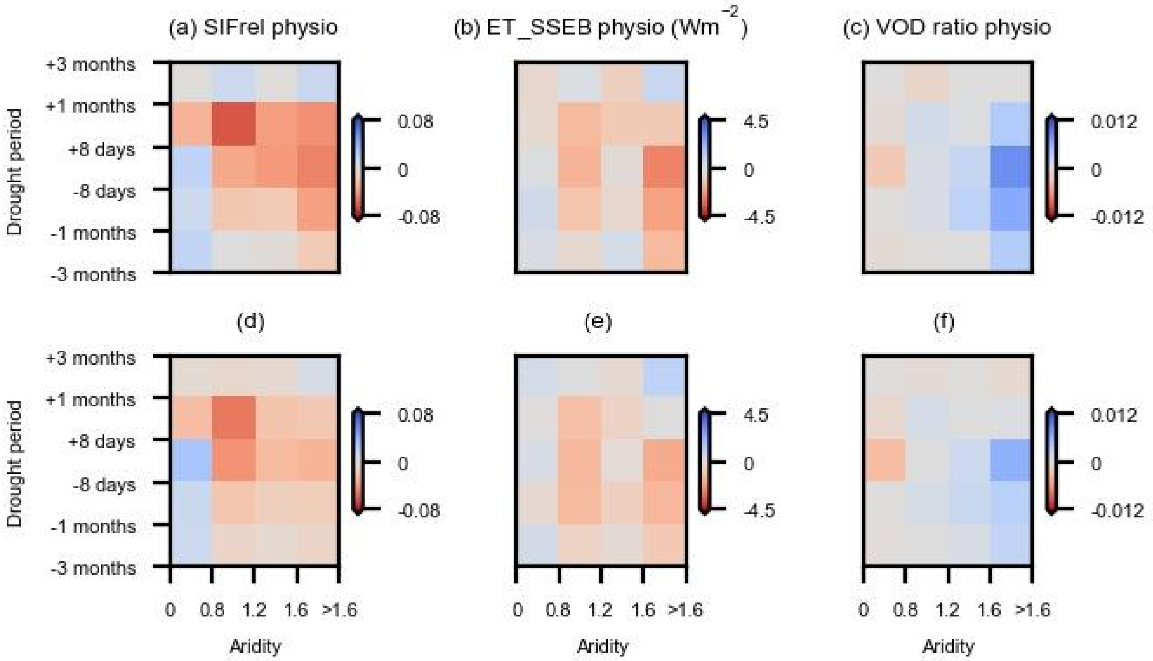

Figure S16. Similar as in Fig.3 (d-f) but disentangling physiological variations using (a-c) SHAP values on random forests and (d-f) multiple linear regression.

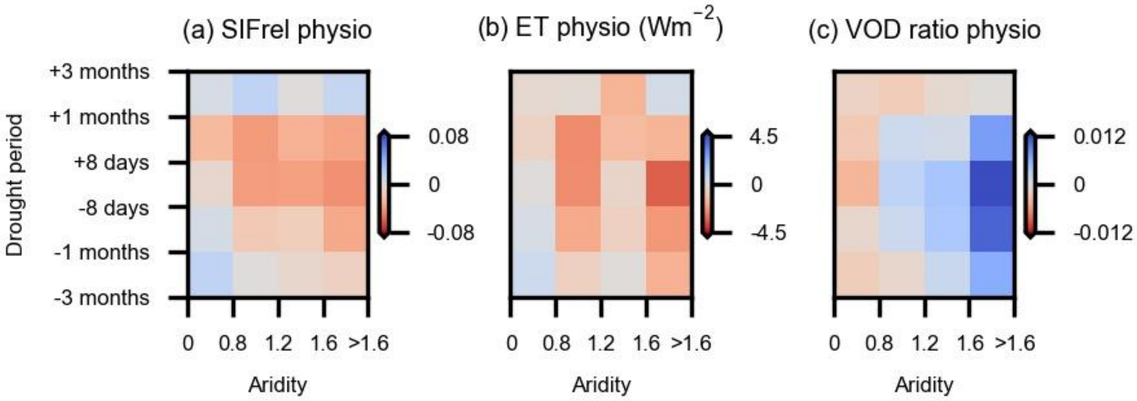

Figure S17. Same as in Fig.3 (d-f) but for regions where the second-strongest drought during the last 40 years is not within the study period 2018–2021. Ecosystem physiology (physio) is estimated as the components of (a) SIFrel, (b) ET, and (c) VOD ratio anomalies remaining after removing the LAI-related variations.

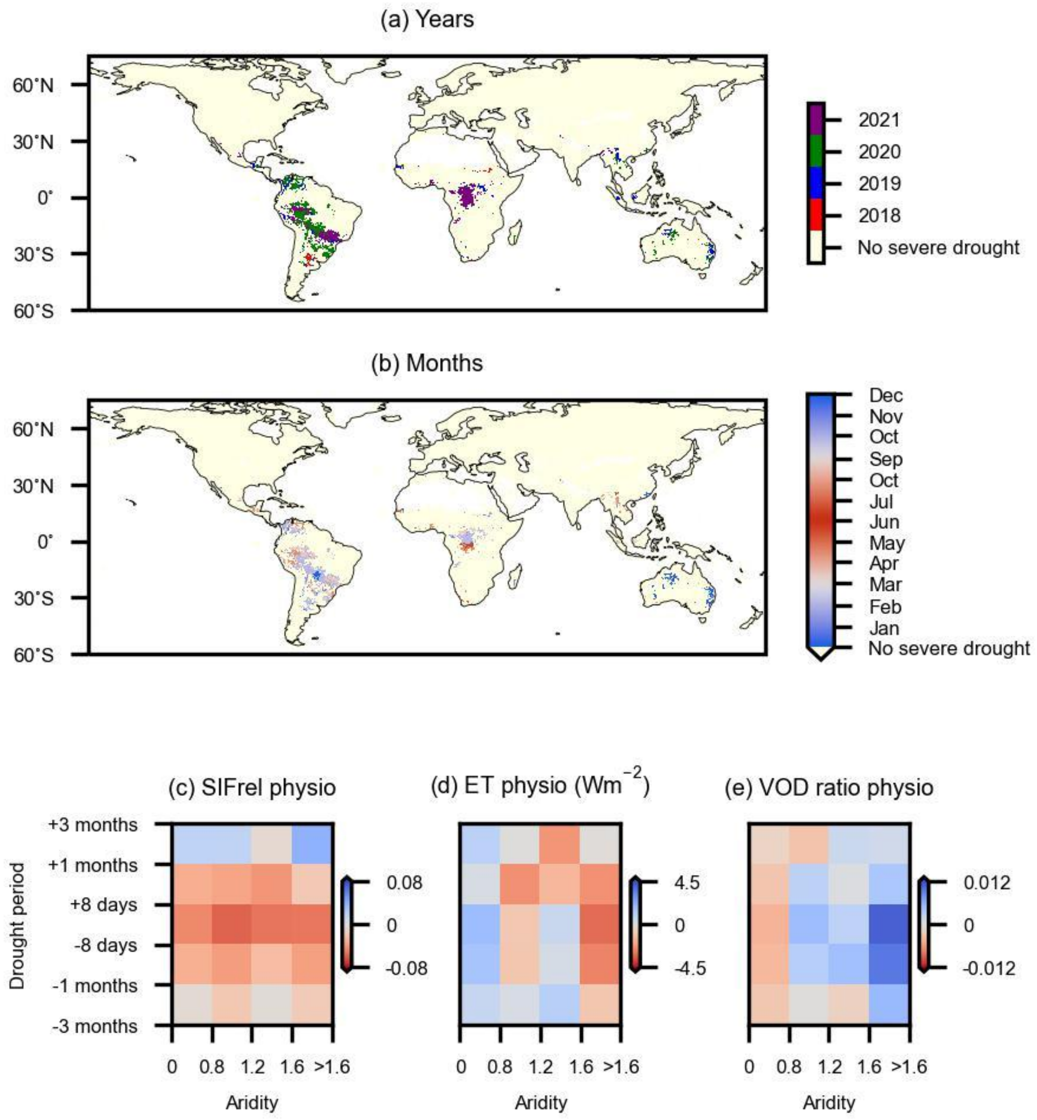

Figure S18. (a, b) Timing of drought peaks, and (c, d, e) vegetation physiological response to drought, but this time we only consider grid cells where the minimum of the 1982-2021 monthly soil moisture is lower than -1.5 standard deviations of soil moisture during the entire period.

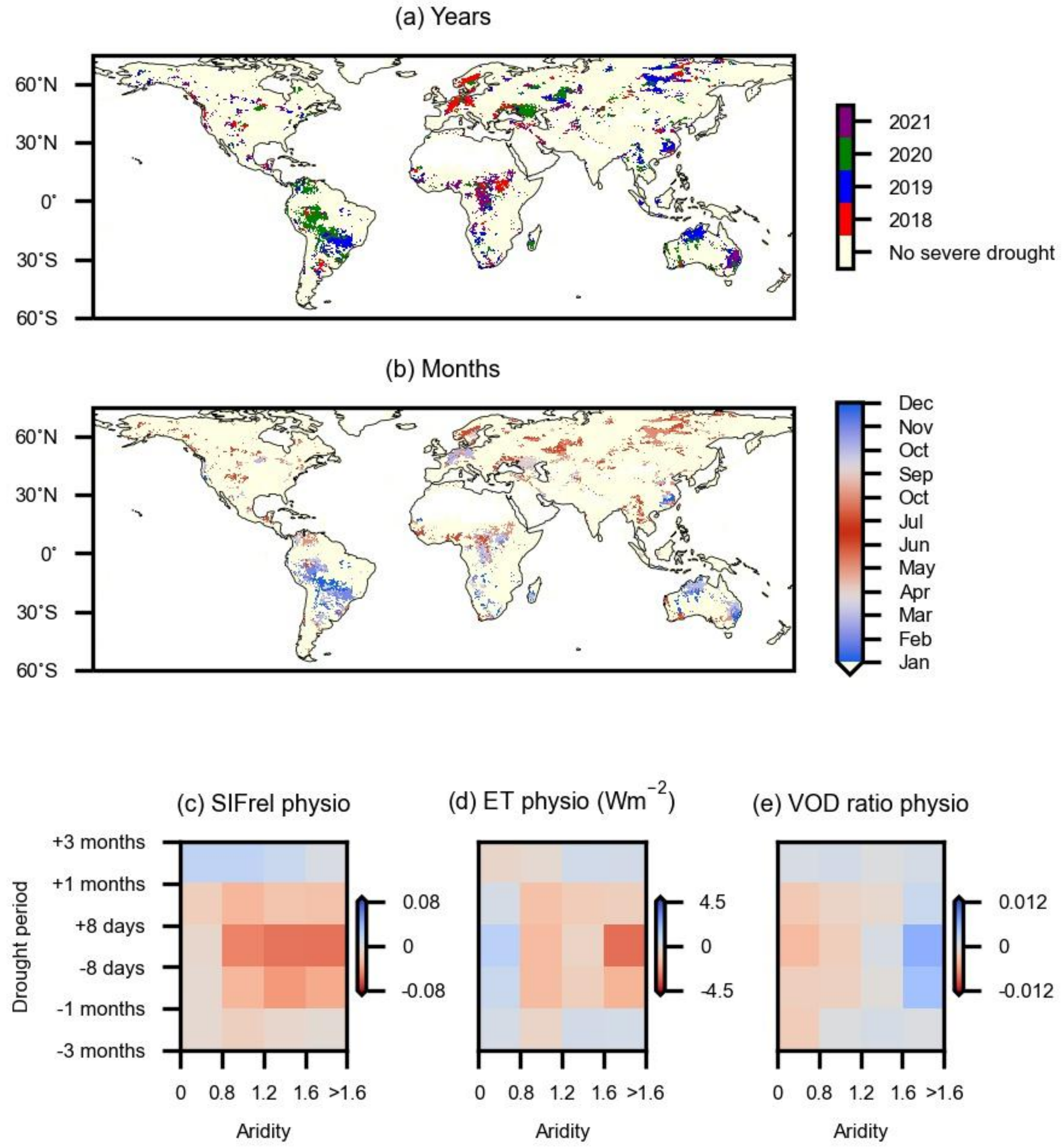

Figure S19. (a, b) Timing of drought peaks similar as in Fig. S2, and (c, d, e) vegetation physiological response to drought similar as in Figure 3 (d-f), but drought peaks are detected by minimum soil moisture anomalies in the growing season.

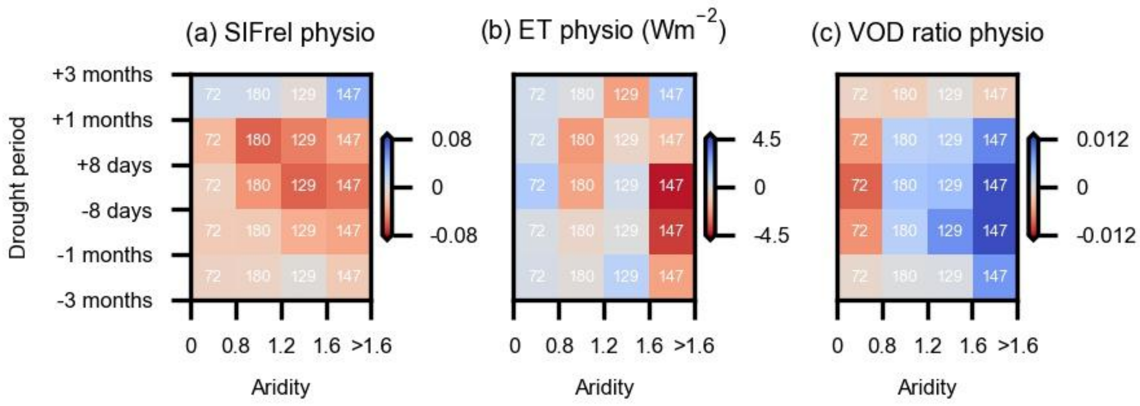

Figure S20. Same as in Fig. 3 (d-f) for observations but focusing on the grid cells selected for SCOPE outputs. Ecosystem physiology (physio) is estimated as the components of (a) SIFrel, (b) ET, and (c) VOD ratio anomalies remaining after removing the LAI-related variations.

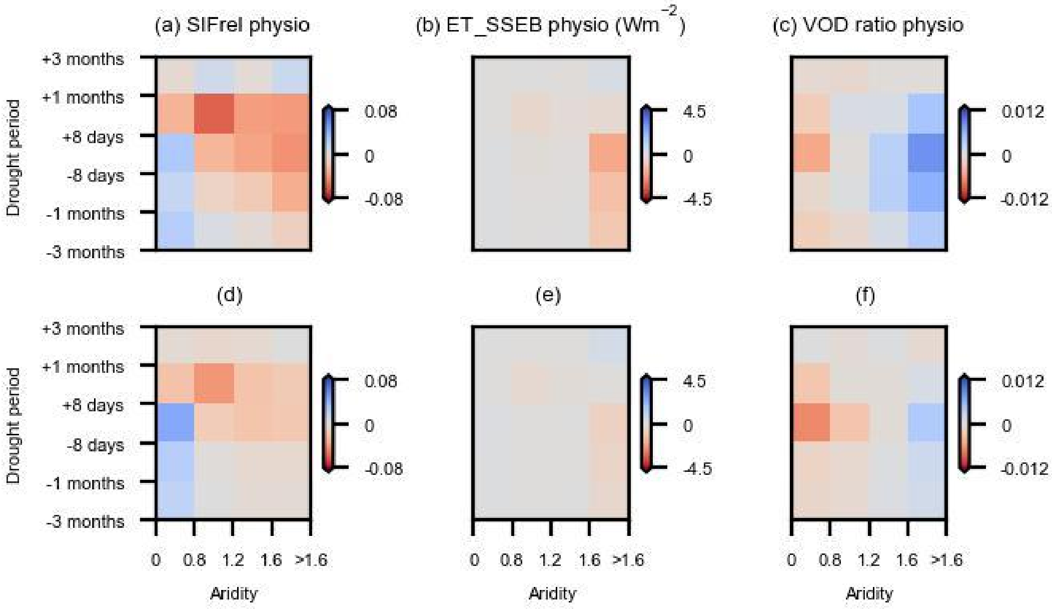

Figure S21. Similar as in Fig. S16 but using only 3 hydro-meteorological variables (temperature, incoming shortwave radiation, and soil moisture) to disentangle physiological variations by (a-c) SHAP values on random forests and (d-f) multiple linear regression.
